# Arterio-Venous Fistula Calcifications—Risk Factors and Clinical Relevance

**DOI:** 10.3390/biomedicines12112464

**Published:** 2024-10-27

**Authors:** Iulia Dana Grosu, Oana Stirbu, Adalbert Schiller, Flaviu Bob

**Affiliations:** 1 Department of Internal Medicine II—Nephrology University Clinic, “Victor Babeș” University of Medicine and Pharmacy, Eftimie Murgu Sq. No. 2, 300041 Timisoara, Romaniabob.flaviu@umft.ro (F.B.); 2 Centre for Molecular Research in Nephrology and Vascular Disease, Faculty of Medicine, “Victor Babes” University of Medicine and Pharmacy, Eftimie Murgu Sq. No. 2, 300041 Timisoara, Romania; 3 County Emergency Hospital, L. Rebreanu Street, Nr. 156, 300723 Timisoara, Romania; 4 B Braun Avitum Dialysis Centre, Cal. Aurel Vlaicu 41-43A, 310141 Arad, Romania; 5 Department of Nephrology and Dialysis, Arad County Hospital, 310158 Arad, Romania

**Keywords:** arterio-venous fistula, hemodialysis, calcifications, arterio-venous fistula complications, ultrasound

## Abstract

(1) Background: Arterio-venous fistulas (AVFs) are considered the gold-standard vascular access (VA) in patients on maintenance hemodialysis (HD) therapy. AVF calcifications represent a less studied VA related complication, even though HD patients are at a higher risk for extraosseous calcifications. The aim of this study is to assess the prevalence and risk factors of AVF calcifications, as well as the 5-year impact on AVF functionality and on overall mortality. (2) Methods: We conducted a 5-year prospective study including 161 patients on maintenance HD therapy. At baseline, we collected data related to VA history, comorbidities, demographics, subjective global assessment scale (SGA), and biochemical parameters. All patients underwent a complete AVF ultrasound and we recorded AVF blood flow and the presence of AVF calcifications, stenoses, and aneurysms. (3) Results: In our study, we found an AVF calcification prevalence of 39%. In a univariate analysis, we found that patients with AVF calcifications were associated with other AVF complications as well (stenoses, aneurysms), had longer AVF and HD vintage, as well as higher serum calcium and PTH values. In a multivariate analysis, we found that patients with a longer HD vintage and higher calcium values were independently associated with AVF calcifications. AVF calcifications did not affect 5-year fistula patency, nor were they associated with a higher mortality risk in our group of patients. (4) Conclusions: AVF calcifications were a frequent finding in our analysis, but their presence does not seem to affect the 5-year AVF patency.

## 1. Introduction

Vascular calcifications (VC) are widely recognized complications of chronic kidney disease (CKD), leading to increased cardiovascular mortality in this population group [1]. The complex pathogenic mechanisms leading to VC have been extensively studied and include CKD associated mineral bone disease [2,3], uremic-milieu related toxins [4], and persistent inflammation [5]. 

Even though vascular calcifications increase as CKD progresses, there is less research performed regarding hemodialysis (HD) vascular access calcifications (VAC), particularly those in arteriovenous fistulas (AVFs).

AVFs are considered the” lifeline” of end stage renal disease (ESRD) patients and the best option for vascular access [6]. Late fistula failure is considered as an important factor related to morbidity in this category of patients [7] and is generally defined as the complete occlusion due to thrombosis or stenosis, occurring after 3 months of use [8]. There are several reports about factors contributing to late fistula failure, including dialysis related factors (hypotension, dialysis vintage, needling technique), uremic milieu related factors (chronic inflammation, mineral-bone disease- CKD-MBD), comorbidities, and concomitant complications [9]. Current vascular access guidelines recommend regular VA monitoring through physical examination, mostly to target complications that require immediate action (such as AVF stenosis or thrombosis). Consequently, ultrasound imaging is recommended to be performed in suspected VA dysfunction as a first line imaging technique, and not routinely, in a scheduled manner [10,11].

AVF Doppler ultrasound (DUS) assessment has the advantage that it may be performed in outpatient HD centers and diagnoses a wide array of VA related complications such as stenosis, thrombosis, aneurysms, steal syndrome, and insufficient maturation [12]. Moreover, AVF DUS surveillance has been reported to improve VA patency due to timely diagnosis of subclinical complications [13]. US techniques can detect vascular calcifications, which appear as hyperechogenic areas with a posterior shadowing. Moreover, US is the preferred technique to diagnose carotid artery calcifications [14] and has a good sensitivity and specificity in the detection of peripheric arterial calcifications (upper and lower extremity) [15]. 

VC occur frequently in CKD patients and are considered not only a consequence of CKD-MBD but also a surrogate indicator of cardiovascular damage. Patients with calcified vessels have a much higher cardiovascular mortality due to arterial stiffening. The pathogenic cascade determining VC involves a dysregulated mineral metabolism due to the upregulation of promoters and inhibition of protective molecules (fetuin- A, matrix Gla protein). Vascular calcifications may be situated either in the intimal layer (involving calcified atheromatous plaques), but also in the medial layer (involving the osteogenic transition of vascular smooth muscle cells) [15]. Even though there are many reports regarding pathogenic mechanisms of arterial calcifications in CKD, there is scarce evidence that they may be valid when considering AVF calcifications. 

Considering the importance of maintaining VA patency and reducing VA complications, the aim of this study is to investigate those factors associated with AVF calcifications and whether the presence of calcifications may reduce the lifespan of the vascular access. Additionally, bearing in mind the cardiovascular burden of VCs, a secondary aim of the current study is to assess whether AVF calcifications had an impact on the patients’ overall 5-year mortality. 

## 2. Materials and Methods

Our prospective, single center 5-year longitudinal study included 205 prevalent patients on maintenance HD therapy. We excluded patients who performed dialysis on arteriovenous grafts (AVGs), *n* = 2 (1%), or on central venous catheters (CVCs), *n* = 42 (20%). Therefore, we obtained a total of 161 patients undergoing HD sessions on a patent, mature AVF. The reason for AVG exclusion was the small number of patients, which leads to lack of statistical significance, whereas the exclusion of patients with CVCs was because our study design sought to perform ultrasound on a functioning AVF. 

All ultrasound examinations were performed during August 2018, and the patients were followed up for 5 years, until August 2023. The data were censored for patient death, transplantation, or AVF patency loss. 

### 2.1. Patient Demographics and Comorbidities

For each patient, we recorded the following variables at the time of ultrasound: age, sex (M/F), HD vintage (months), AVF vintage (months from creation), and AVF type (radiocephalic- RC, brachio-cephalic BC, brachio-basilic- BB). The VA history we recorded included whether the current AVF was the first VA and whether the patients had previous ipsilateral central venous catheters (CVCs). Patients’ comorbidities were recorded, including the presence of diabetes mellitus and the presence of cardiac valve calcifications. Cardiac valve calcification assessment was performed via echocardiography during the month of AVF US evaluation using the same ultrasound machine but using a 2–5 MHz transducer. 

All procedures were carried out in accordance with the ethical standards of the institutional research committee and in line with the Declaration of Helsinki, and the study was authorized by the Dialysis Center’s Ethics Committee of BBRAUN Dialysis Centers nr. 33/30.06.2021. Prior to any study procedure being conducted, eligible patients were asked to provide a written informed consent.

AVF Doppler ultrasound was performed on all patients enrolled in the study in August 2018 by a team of two senior nephrologists with VA ultrasound experience, using an Edan Acclarix AX4 ultrasound machine equipped with a linear transducer 7–12 MHz. The following steps were employed in the US protocol, as per current AVF US assessment recommendations. 

### 2.2. Ultrasound Examination

1. Measurement of brachial artery blood flow—regardless of the AVF type, blood flow was calculated in the brachial artery, upstream of its division, as per the Standardized Duplex US Methodology for AVF evaluation [16]. The artery was observed in a B-mode longitudinal section, then color-mode and Doppler mode were implied. The Doppler waveform of the feeding artery was assessed as per current recommendations [17]. The sample volume encompassed approximately 75% of the vessel caliber and the insonation angle was set below 60 degrees. The arterial diameter was measured intima to intima. The blood flow was calculated by the US machine software in mL/min.
2. Evaluation of the anastomosis (mm).
3. Evaluation of the draining vein—using B-mode, Color, and Doppler. The presence of AVF stenosis, calcifications, and aneurysms was recorded. The following definitions were used for each complication, as per current guidelines [10,11]:

AVF stenosis criteria considered were those set by the Prague group [18]: diameter reduction by 50%, peak systolic velocity increase in the stenotic region by 2–3 times the PSV in the pre-stenotic region, and the additional criterion of a residual diameter below 2 mm. 

AVF aneurysms were defined as the presence of localized, enlarged areas of the AVF, to diameters of over 18 mm [19].

AVF calcifications were defined as the presence of hyperechogenic areas adjacent to the AVF wall, with or without posterior wall shadowing. The determination of AVF calcifications in our study was qualitative and we subdivided their localization into juxta-anastomotic (maximum 2 cm from the anastomosis site), distal (the rest of the AVF), or both.

### 2.3. Biochemical Analysis

In the month of AVF ultrasound assessments we performed, the following hematological and biochemical tests were conducted: complete blood cell count including hemoglobin (Hb—g/dL), serum calcium (Ca—mg/dL), phosphorus (P mg/dL), Bicarbonate (mmol/L), ferritin (mmol/L), iPTH (pg/mL), albumin (g/dL). For the assessment of patients’ nutritional status, we also recorded the SGA (Subjective Global Assessment) score, which consists of the following items: weight changes, dietary intake, gastrointestinal symptoms, functional capacity, comorbidities, and physical exams, including signs of muscle wasting and decreased fat stores. 

### 2.4. Statistical Analysis

Statistical analysis was performed using the MedCalc 22.014 software. We performed the Kolmogrov–Smirnov test to assess the normality of the data distribution. For normally distributed continuous variables, we reported the mean +/− standard deviation, while for non-normal variables we reported medians and interquartile ranges. Categorical variables were expressed in percentages. Comparisons between the variables were performed using the Chi-square test (for categorical variables) and the Mann–Whitney test (for quantitative non-normally distributed variables). The relationship between the presence of calcifications and the baseline variables was assessed using binary logistic regression. We used the Cox proportional hazards model to determine hazard ratios and AVF survival curves as well as influence on overall mortality according to the presence of calcifications. We considered a value below *p* < 0.05 as statistically significant. 

## 3. Results

The present analysis included 161 patients, mean age 57.8 +/− 12.9 years, with a higher male prevalence (67%), undergoing chronic hemodialysis sessions for more than 3 months. We also recorded the type of AVF (radio-cephalic—RC, brachio-cephalic—BC, brachio-basilic—BB) and noted there were no differences between the two groups. Table 1 describes the comparative data of the patients according to the presence of vascular calcifications. We noted a prevalence of 39% of AVFs with calcifications. We noted that 78% (*n* = 49) had juxta-anastomotic calcifications, 19% (*n* = 12) had distal calcifications, and 3% (*n* = 2) had both. 

The group with VA calcifications had a longer HD vintage (*p* < 0.001) and a longer AVF vintage (*p* < 0.001). Moreover, at baseline, the patients with VA calcifications were associated more frequently with concomitant AVF complications (aneurysms- *p* < 0.001 and stenosis *p* = 0.02), higher PTH levels (*p* = 0.03), and higher ekT/v values (*p* = 0.001). 

In a univariate logistic regression model, we found that AVF vintage, HD vintage, the presence of concomitant stenosis and aneurysms, higher calcium, iPTH, and Hb levels, as well as higher AVF blood flow were associated with AVF calcifications.

When we performed a multivariate logistic regression model, the only independent predictors for the development of AVF calcifications were higher calcium levels (*p* = 0.007) and the HD vintage (*p* = 0.03) (Table 2).

**Figure 1:**
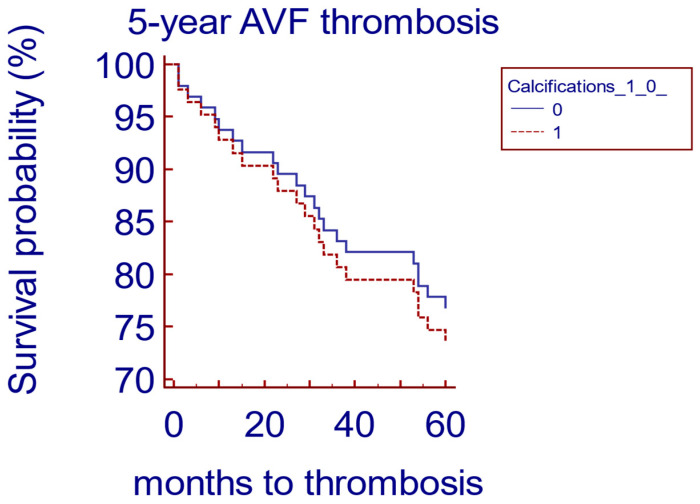
Cox proportional hazards model of patients with AVF calcifications versus patients without AVF calcifications.

At the 5- year follow-up, we found that 41% of the AVFs in the group with calcifications were still functioning, as compared to only 29% of the AVFs in the group without calcifications (Table 3). We performed a Cox proportional hazards model, censored for death and transplantation, and we found that the presence of AVF calcifications did not represent a risk factor for 5-year AVF failure, with a HR of 0.15, 95% CI 0.5030 to 2.6956, *p* = 0.72 (Figure 1).

**Figure 2:**
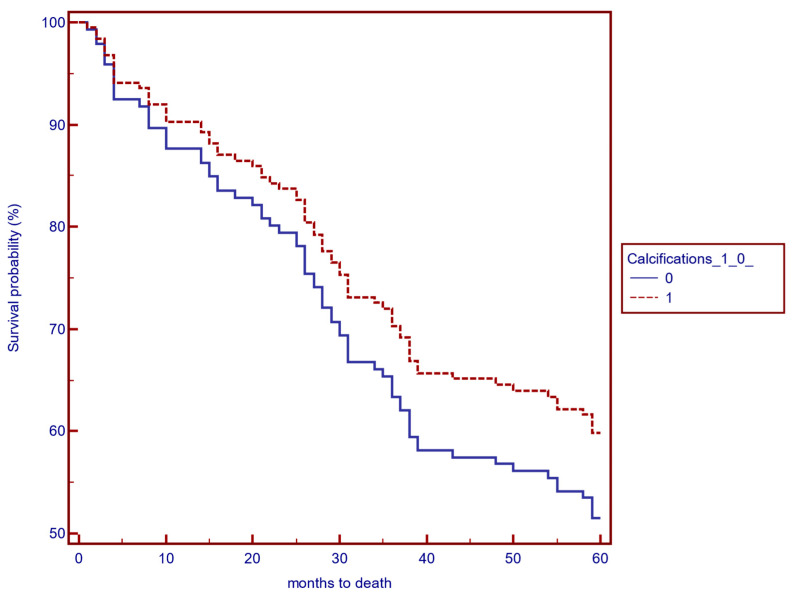
Cox proportional hazards model—5-year survival analysis of patients with versus without AVF calcifications.

The presence of AVF calcifications as a risk factor for mortality was assessed using a Cox proportional hazards model. The present study shows that patients with AVF calcifications did not exhibit a higher risk of mortality—HR 0.1, 95% CI 7.36–640, *p* = 0.96. (Figure 2). 

## 4. Discussion

Diffuse vascular calcifications are a frequent finding in patients with ESRD, and they have been recognized as part of the clinical picture of CKD-associated mineral bone disorder (CKD-MBD) [20]. Even though arterial vascular calcifications have been incriminated as a risk factor for cardiovascular mortality in CKD [21], the prevalence and clinical relevance of AVF calcifications has been much less studied. In our current study, we investigated the risk factors associated with AVF calcifications detected by routine US examination, and whether this finding is associated both with lower AVF survival rates and with overall lower patient survival.

In the present study, we reported a prevalence of 39% of AVF calcifications using US, higher than those reported by Schlieper et al. [22], who used two-dimensional X rays, and found a prevalence of 23%, and by Toussaint [23], who used CT fistulograms and found a prevalence of only 14%. US evaluations are much easier to perform, often using a hand-held or portable device available in the HD center, as compared to radiological investigations. The report of Wang et al. [24] concluded that ultrasound is more sensitive in detecting vascular calcifications than conventional radiology for upper extremity arteries. Moreover, ultrasound is the first-line technique recommended for carotid plaque calcifications [25] and has an established role in diagnosing calcified peripheral artery disease lesions [26].

The pathogenesis of vascular calcifications in CKD have been broadly studied, especially regarding their arterial involvement. This includes the imbalance between calcification promoters and calcification inhibitors. The key player is represented by vascular smooth muscle cells, which are very sensitive to osteogenic molecules such as bone morphogenetic protein-2, osteopontin, and matrix γ-carboxyglutamic acid protein, Fetuin-A, or Klotho, which are upregulated in the setting of CKD. Several studies have been conducted regarding the arteries prior to vascular access creation. Arteries have varying degrees of calcium deposits, especially in the medial layer, prior to AVF formation [15,27]. Micro-calcifications in the inflow artery may predispose newly created AVFs to lack of maturation, according to Lee et al. [28]. Additionally, an ultrasonographic marker for identifying useful calcified arteries prior to AVF surgery is the resistance index (RI) during reactive hyperemia. A recent study [29] showed that the presence of calcified arteries, but with a suitable diameter and an RI of up to 0.9, is associated with good AVF patency. Moreover, there has been a proposed ultrasound classification of arterial calcifications as “mild” (hyperechogenic arterial walls with white spots), moderate (“patchy calcifications”), and severe (continuous calcifications with severe distal shadowing). 

The impact of preexisting arterial changes on AVF maturation has been also approached by Allon et al. [30]. In contrast to the work of Lee et al. [28], they have showed that even though histologically proven fibrosis and microcalcifications in the feeding artery are frequent, this does not account for AVF non-maturation. In another study performed by the same author [31] on a larger cohort of patients, a similar result was obtained: there was no correlation between preexisting micro-calcifications and subsequent AVF stenosis or insufficient development. 

Venous calcifications have been less studied, and there are no clear associations between calcifications or hyperplasia and primary AVF failure. Moreover, vascular smooth muscle cells accumulate predominantly in arteries versus veins [32], which might suggest different pathogenic mechanisms involved in venous calcifications. 

With our current study, we aimed to find clinical associations that potentially highlight underlying mechanisms regarding arteriovenous fistula calcifications. In the univariate analysis, patients with calcified AVFs had been undergoing HD for longer and had a longer vintage of their VA. Similar results have been obtained by Roca-Tey et al. [33], using CT scanning as the method for calcification detection. This might explain why these AVFs presented with additional complications, such as stenoses or aneurysms. 

In a comparative study regarding AVF ultrasound features, Wang et al. [24] showed that AVF calcifications were more frequent in stenotic AVFs, which had a poorer revascularization prognosis after percutaneous transluminal angioplasty. However, our study shows that patients with calcified AVFs had higher eKT/v values and higher Qa values, even though the latter parameter did not reach statistical significance (*p* = 0.05). According to the KDOQI guidelines [34], the eKT/v target value should be above 1.2 to consider a minimally adequate HD session. A crucial role in dialysis adequacy is represented by the blood flow provided by the AVF (Qa), which should be above 600 mL/min. Qa tends to increase over time; however, it is widely variable from patient to patient due to anatomical and surgical particularities, leading to different levels of shear stress in the vessel wall. AVF geometric parameters (anastomosis angles, tortuosity, collateral veins) play an important part in the development of mechanical stress and have been linked to AVF complications, as demonstrated by He et al. [35]. 

In our study, we also analyzed the concomitance of cardiac valve calcifications and we found no associations with AVF calcifications. Cardiac valve calcifications are common in HD patients and occur because of CKD associated mineral-bone disorder (CKD-MBD). The prevalence of aortic and mitral valve calcifications is reported to be four times higher than in non-CKD patients [36]. Moreover, the presence of cardiac calcifications, especially mitral, is considered an independent risk factor for all-cause mortality [37]. Moreover, the process is considerably more accelerated when compared to the general population due to the implication of multiple pathogenic mechanisms. Thus, similarly to CKD associated vascular calcifications, the pathogenesis involves traditional risk factors (diabetes mellitus type 2, arterial hypertension, obesity, tobacco use, age), but mainly focuses on the changes in mineral bone metabolism, uremic milieu, and chronic inflammation. The changes that occur in CKD involve a high calcium x phosphorus product leading to increased fibroblast growth factor 23 (FGF23), low vitamin D levels, and high parathormone levels (PTH) [38]. Literature data regarding the treatment targeting CKD-MBD and vascular calcifications are vast and conflicting. Current KDIGO guidelines [20] do not recommend phosphorus-lowering medications such as calcium-based phosphate binders or sevelamer as preventative measures for vascular calcifications. Reducing PTH levels has proven beneficial on all-cause mortality, as described in a study by Lamina et al. [39]. Current KDIGO guidelines [20] recommend treating hyperparathyroidism with either calcimimetics, calcitriol, or vitamin D analogues based on calcium and phosphorus levels, as well as clinical particularities. Paricalcitol and calcitriol have shown beneficial effects on vascular calcifications and increased survival in the dialysis population [40], even though there are no randomized control trials to demonstrate this. Cinacalcet, associated with vitamin D therapy, has reduced cardiac and vascular calcification both in experimental models [41] and in dialysis patients [42]. However, all studies involved arterial and cardiac valvular structures, and it is not known whether there is an overlap with venous calcifications. 

Therefore, in patients with CKD-MBD, vascular smooth muscle cells present in the endothelial structures suffer an osteogenic transformation. The disposition of vascular smooth muscle cells occurs in most vessels, but veins have more loosely organized smooth muscle cells. A recent study by Rojas et al. [43] showed how different the response to different hemodynamic or biochemical stimuli occurs in arteries versus veins. The fact that AVFs have a different underlying tissue might explain why they do not respond in the same manner to calcification triggers as arterial endothelial cells. Studies regarding the cross-talk between calcifications in the venous versus arterial system are scarce; however, Schlieper et al. [22] performed a similar analysis to ours and found no relation between carotid or valvular calcifications and AVF calcifications. In the multivariate analysis, our study found that calcium values were predictors for AVF calcifications. This has not been confirmed by previous analyses [22,44].

An additional aspect that was included in the present research was the assessment of inflammation (CRP levels), but also malnutrition, through the subjective global assessment (SGA score). Malnutrition-inflammation-atherosclerosis (MIA) syndrome is a frequent finding in HD patients, contributing to their increased mortality risk [45]. It is associated with persistent inflammation, decreased protein synthesis, and low albumin levels, as well as sarcopenia. Moreover, it has been found to be an additional mechanism involved in CKD-MBD and accelerated atherosclerosis [46]. In the clinical setting, the subjective global assessment scale is a validated and reliable test to assess nutritional status in HD patients [47]. In our study we did not find any associations between an increased SGA score (reflecting MIA syndrome) and AVF calcifications. Similar results were obtained in previous studies, but only considering biochemical factors. [22,33].

Our study showed no difference in 5-year AVF survival rates in the group with or without calcifications. Roca Tey et al. have evaluated AVF calcifications using spiral CT scans and have classified different extents of calcifications [33], but, similarly to our result, have found that AVF functionality is not affected by any degree of calcification. This result is different from those obtained by Jankovic [44], who found that calcified AVFs (assessed on arm X-rays) have a 3.4 risk of to develop 5-year failure (*p* = 0.049). We did not obtain a significant correlation regarding the influence of AVF calcifications on mortality. By contrast, Schlieper obtained a 2.13 risk of mortality if AVF calcifications (assessed on arm X-rays) were present [22]. Therefore, all the studies presented are monocentric, use different methods of assessment (X-ray or CT scan) and have heterogenous results. To our knowledge, this is the first study using ultrasonography whereas AVF calcifications are concerned. This aspect is of clinical importance due to the more extensive use of ultrasound in the screening of AVF complications, rather than radiological examinations. Our study group included a small number of participants; therefore, we believe that we cannot draw firm conclusions regarding the relevance of AVF calcifications as a risk factor for overall mortality. However, the associations that we have encountered suggest that AVF calcifications should not be regarded as detrimental as arterial or valvular calcifications. More studies are, nevertheless, needed to prove this hypothesis.

The present study has several limitations. Firstly, we have not included the use of CKD-MBD medication in our analysis. This might impact the outcomes related to CKD-MBD associated parameters, and might modulate the extent of the AVF or valvular calcifications, as described previously. Secondly, the ultrasound technique, although easy to perform even in a bedside setting, is operator dependent and unquantifiable. In our study, we performed a qualitative assessment, but we acknowledge that a quantitative or even semi-quantitative assessment would be more accurate for research purposes. The utility of assessing the exact severity of AVF calcifications using US methods requires further validation. Third, we have not considered AVF geometry issues (tortuosity, collateralizations), which may influence the blood flow non-linearity and contribute to the calcification process. 

## 5. Conclusions

In conclusion, AVF calcifications are a frequent finding on routine ultrasound examinations (39% of patients) and are associated with other AVF complications (stenoses, aneurysms). We found that AVF calcifications correlate with calcium values but not with other routinely assessed parameters. Even though they are linked to HD vintage, our study has shown that they do not affect the 5-year AVF survival. Moreover, in our study lot, AVF calcifications did not contribute to the patients’ higher mortality risk. 

## Figures and Tables

**Table 1:** Comparative data of the patients according to the presence of AVF calcifications (HD—hemodialysis, CVC = central venous catheter, AVF = arterio-venous fistula, Qa = blood flow, eKt/v = e—estimated *K*—dialyzer clearance of urea, *t*—dialysis time, *V*—volume of distribution of urea, Hb = hemoglobin, Ca = serum calcium, iPTH = intact parathyroid hormone).

Variable	All (*n* = 161)	With Calcifications (*n* = 63)	Without Calcifications (*n* = 98)	*p*
Age (median, IQR)	57.8 +/− 12.9	54.7 +/− 12.3	59.8 +/− 12.9	0.01
AVF type (RC/BC/BB)	78 (48.4%)	31 (49.2%)	47 (47.9%)	0.8
	67 (41.6%)	25 (39.6%)	42 (42.8%)	0.6
	18 (10%)	7 (12.2%)	9 (9.3%)	0.5
HD vintage (months)	48 (16.5–83.5)	86.5 (61–128)	20 (9–53)	<0.001
AVF vintage at US evaluation (months)	36 (9.8–70.3)	72 (40–104)	14.5 (6–42)	<0.001
First vascular access (number, %)	18 (11.2%)	11 (17.5%)	7 (7.1%)	0.04
Sex (F/M)	53 F, 108 M (33%, 67%)	18 F, 45 M (29%, 71%)	35 F, 64 M (36%, 64%)	0.3
Diabetes mellitus (number)	38	14	24	0.74
Valvular calcifications (number)	55	19	36	0.39
Previous ipsilateral CVC (1/0)	65	26	39	0.72
Concomittant Aneurysm (1/0)	52	31	21	0.0002
Concomittant stenosis (1/0)	23	14	9	0.02
Qa (ml/min)	783 (530–1289)	800 (581–1626)	750 (504–1181)	0.05
eKt/v < 1.2 (number)	40	10	30	0.03
eKt/v	1.21 (1.31–1.44)	1.39 (1.25–1.5)	1.27 (1.17–1.42)	0.001
Subjective Global Assessment (SGA)	3 (2–4)	3 (2–3)	3 (2–6)	0.12
Hb (g/dL)	10.8 +/− 1.5	11.1 +/− 1.45	10.6 +/− 1.54	0.04
Ca (mg/dL)	8.9 +/− 0.7	9.16 +/− 0.8	8.83 +/− 0.67	0.005
Phosphorus (mg/dL)	4.7 (3.89–5.67)	4.93 (4.03–6.19)	4.45 (3.78–5.41)	0.07
Bicarbonate (mmol/L)	19.8 +/− 2.1	19.8 +/− 2.2	19.7 +/− 2.2	0.78
Ferritin (mg/dL)	501 (356–689)	548 (358–799)	484 (343–688)	0.4
iPTH (pg/mL)	330 (149–838)	520 (162–1041)	280 (137–556)	0.03
Albumin (g/dL)	4.1 (3.94–4.39)	4.24 (4.06–4.42)	4.09 (4.87–4.27)	0.05

**Table 2:** Multivariate logistic regression (Ca = calcium, HD = hemodialysis, iPTH = intact parathyroid hormone, eKt/v = e—estimated *K*—dialyzer clearance of urea, *t*—dialysis time, *V*—volume of distribution of urea).

Variable	Coefficient	Std. Error	*p*
Age (years)	−0.003818	0.002910	0.1918
Albumin (g/dL)	−0.02119	0.09377	0.8215
Aneurysms (1/0)	0.07299	0.07694	0.3446
AVF vintage (months)	0.001798	0.001652	0.2784
Ca (mg/dL)	0.1317	0.04809	0.0070
Hb	0.01645	0.02345	0.4844
HD vintage (months)	0.003158	0.001476	0.0343
iPTH (pg/mL)	0.00001751	0.00006295	0.7813
Qa	0.00004139	0.00003158	0.1923
Phosphate (mg/dL)	0.02122	0.02393	0.3769
Stenosis (1/0)	0.1541	0.09738	0.1161
eKt/V	0.01082	0.07933	0.8917

**Table 3:** Patients with or without AVF calcifications with functioning AVFs at the beginning of the study and at the 5-year follow-up.

Number of Patients with a Functional AVF	Functioning AVFs—Beginning of the Study (*n*)	Functioning AVFs—5 Year Follow-Up (*n*)	Functioning AVFs—5 Year Follow-Up (%)
Group with AVF calcifications	63	26	41%
Group without AVF calcifications	161	46	29%

## Data Availability

The raw data supporting the conclusions of this article will be made available by the authors on request.

## References

[1] De Jager D.J., Grootendorst D.C., Jager K.J., van Dijk P.C., Tomas L.M.J., Ansell D., Collart F., Finne P., Heaf J.G., De Meester J. (2009). Cardiovascular and Noncardiovascular Mortality Among Patients Starting Dialysis. JAMA.

[2] Jablonski K.L., Chonchol M. (2013). Vascular calcification in end-stage renal disease. Hemodial. Int..

[3] Valdivielso J.M. (2011). Calcificación vascular: Tipos y mecanismos [Vascular calcification: Types and mechanisms]. Nefrologia.

[4] Kyriakidis N.C., Cobo G., Dai L., Lindholm B., Stenvinkel P. (2021). Role of Uremic Toxins in Early Vascular Ageing and Calcification. Toxins.

[5] Viegas C., Araújo N., Marreiros C., Simes D. (2019). The interplay between mineral metabolism, vascular calcification and inflammation in Chronic Kidney Disease (CKD): Challenging old concepts with new facts. Aging.

[6] Santoro D., Benedetto F., Mondello P., Pipitò N., Barillà D., Spinelli F., Ricciardi C.A., Cernaro V., Buemi M. (2014). Vascular access for hemodialysis: Current perspectives. Int. J. Nephrol. Renovasc Dis..

[7] Girerd S., Girerd N., Frimat L., Holdaas H., Jardine A.G., Schmieder R.E., Fellström B., Settembre N., Malikov S., Rossignol P. (2020). Arteriovenous fistula thrombosis is associated with increased all-cause and cardiovascular mortality in haemodialysis patients from the AURORA trial. Clin. Kidney J..

[8] Yen C.C., Tsai C.F., Luo Y.Y., Yang H.-Y., Liu M.-Y., Hung P.-H., Hsu Y.-H. (2018). Factors affecting fistula failure in patients on chronic hemodialysis: A population–based case–control study. BMC Nephrol..

[9] Smith G.E., Gohil R., Chetter I.C. (2012). Factors affecting the patency of arteriovenous fistulas for dialysis access. J. Vasc. Surg..

[10] Schmidli J., Widmer M.K., Basile C., de Donato G., Gallieni M., Gibbons C.P., Haage P., Hamilton G., Hedin U., Kamper L. (2018). Editor’s Choice-Vascular Access: 2018 Clinical Practice Guidelines of the European Society for Vascular Surgery (ESVS). Eur. J. Vasc. Endovasc. Surg..

[11] Lok C.E., Huber T.S., Lee T., Shenoy S., Yevzlin A.S., Abreo K., Allon M., Asif A., Astor B.C., Glickman M.H. (2020). National Kidney Foundation. KDOQI Clinical Practice Guideline for Vascular Access: 2019 Update. Am. J. Kidney Dis..

[12] Meola M., Marciello A., Di Salle G., Petrucci I. (2021). Ultrasound evaluation of access complications: Thrombosis, aneurysms, pseudoaneurysms and infections. J. Vasc. Access..

[13] Khairy A., Rashed A.M., Shahat M., Ali H. (2022). The role of regular surveillance on maintenance of patency of vascular access. Egypt. J. Surg..

[14] Del Brutto V.J., Gornik H.L., Rundek T. (2020). Why are we still debating criteria for carotid artery stenosis?. Ann. Transl. Med..

[15] Lanzer P., Hannan F.M., Lanzer J.D., Janzen J., Raggi P., Furniss D., Schuchardt M., Thakker R., Fok P.W., Saez-Rodriguez J. (2021). Medial Arterial Calcification: JACC State-of-the-Art Review. J. Am. Coll. Cardiol..

[16] Teodorescu V., Gustavson S., Schanzer H. (2012). Duplex ultrasound evaluation of hemodialysis access: A detailed protocol. Int. J. Nephrol..

[17] Kim E.S.H., Sharma A.M., Scissons R., Dawson D., Eberhardt R.T., Gerhard-Herman M., Hughes J.P., Knight S., Kupinski A.M., Mahe G. (2020). Interpretation of peripheral arterial and venous Doppler waveforms: A Consensus Statement from the Society for Vascular Medicine and Society for Vascular Ultrasound. Vasc. Med..

[18] Malik J., de Bont C., Valerianova A., Krupickova Z., Novakova L. (2022). Arteriovenous Hemodialysis Access Stenosis Diagnosed by Duplex Doppler Ultrasonography: A Review. Diagnostics.

[19] Mudoni A., Cornacchiari M., Gallieni M., Guastoni C., McGrogan D., Logias F., Ferramosca E., Mereghetti M., Inston N. (2015). Aneurysms and pseudoaneurysms in dialysis access. Clin. Kidney J..

[20] Wheeler D.C., Winkelmayer W.C. (2017). KDIGO 2017 Clinical Practice Guideline Update for the Diagnosis, Evaluation, Prevention, and Treatment of Chronic Kidney Disease-Mineral and Bone Disorder (CKD-MBD). Kidney Int. Suppl..

[21] Yamada S., Nakano T. (2023). Role of Chronic Kidney Disease (CKD)-Mineral and Bone Disorder (MBD) in the Pathogenesis of Cardiovascular Disease in CKD. J. Atheroscler. Thromb..

[22] Schlieper G., Krüger T., Djuric Z., Damjanovic T., Markovic N., Schurgers L.J., Brandenburg V.M., Westenfeld R., Dimkovic S., Ketteler M. (2008). Vascular access calcification predicts mortality in hemodialysis patients. Kidney Int..

[23] Toussaint N.D., Lau K.K., Polkinghorne K.R., Kerr P.G. (2007). Measurement of vascular calcification using CT fistulograms. Nephrol. Dial. Transplant..

[24] Wang Y., Huang X mei Zhang Y., Li J., Li J., Ye Z., Xu L. (2024). Comparison of ultrasound features and lesion sites in dysfunctional arteriovenous fistula. Ren. Fail..

[25] Johri A.M., Nambi V., Naqvi T.Z., Feinstein S.B., Kim E.S.H., Park M.M., Becher H., Sillesen H. (2020). Recommendations for the Assessment of Carotid Arterial Plaque by Ultrasound for the Characterization of Atherosclerosis and Evaluation of Cardiovascular Risk: From the American Society of Echocardiography. J. Am. Soc. Echocardiogr..

[26] Huthart S., Oates C., Allen J., Fiaschi K., Sims A.J., Stansby G. (2022). Validation of a Standardised Duplex Ultrasound Classification System for the Reporting and Grading of Peripheral Arterial Disease. Eur. J. Vasc. Endovasc. Surg..

[27] Pajek J., Malovrh M. (2017). Preoperative ultrasound still valuable for radio-cephalic arteriovenous fistula creation?. J. Vasc. Access.

[28] Lee J.Y., Kim Y.O. (2017). Pre-existing arterial pathologic changes affecting arteriovenous fistula patency and cardiovascular mortality in hemodialysis patients. Korean J. Intern. Med..

[29] Gubensek J. (2024). Doppler ultrasound assessment of calcified radial arteries prior to radio-cephalic arterio-venous fistula placement: An observational study. J. Vasc. Access..

[30] Allon M., Litovsky S., Young C.J., Deierhoi M.H., Goodman J., Hanaway M., Lockhart M.E., Robbin M.L. (2011). Medial fibrosis, vascular calcification, intimal hyperplasia, and arteriovenous fistula maturation. Am. J. Kidney Dis..

[31] Allon M., Robbin M.L., Umphrey H.R., Young C.J., Deierhoi M.H., Goodman J., Hanaway M., Lockhart M.E., Barker-Finkel J., Litovsky S. (2015). Preoperative arterial microcalcification and clinical outcomes of arteriovenous fistulas for hemodialysis. Am. J. Kidney Dis..

[32] Ashraf J.V., Al Haj Zen A. (2021). Role of Vascular Smooth Muscle Cell Phenotype Switching in Arteriogenesis. Int. J. Mol. Sci..

[33] Roca-Tey R., Páez R., Rivas A., Samon R., Ibrik O., Giménez I., Viladoms J. (2009). Prevalence and functional effect of arteriovenous fistula calcifications, evaluated by spiral CT in chronic haemodialysis patients. Nefrologia.

[34] National Kidney Foundation (2015). KDOQI Clinical Practice Guideline for Hemodialysis Adequacy: 2015 update. Am. J. Kidney Dis..

[35] He Y., Northrup H., Roy-Chaudhury P., Cheung A.K., Berceli S.A., Shiu Y.T. (2021). Analyses of hemodialysis arteriovenous fistula geometric configuration and its associations with maturation and reintervention. J. Vasc. Surg..

[36] Rong S., Qiu X., Jin X., Shang M., Huang Y., Tang Z., Yuan W. (2018). Risk factors for heart valve calcification in chronic kidney disease. Medicine.

[37] Raggi P., Bellasi A., Gamboa C., Ferramosca E., Ratti C., Block G.A., Muntner P. (2011). All-cause mortality in hemodialysis patients with heart valve calcification. Clin. J. Am. Soc. Nephrol..

[38] Waziri B., Duarte R., Naicker S. (2019). Chronic Kidney Disease-Mineral and Bone Disorder (CKD-MBD): Current Perspectives. Int. J. Nephrol. Renov. Dis..

[39] Lamina C., Kronenberg F., Stenvinkel P., Froissart M., Forer L., Schönherr S., Wheeler D.C., Eckardt K.-U., Floege J. (2020). Association of changes in bone mineral parameters with mortality in haemodialysis patients: Insights from the ARO cohort. Nephrol. Dial. Transplant..

[40] Cardús A., Panizo S., Parisi E., Fernandez E., Valdivielso J.M. (2007). Differential effects of vitamin D analogs on vascular calcification. J. Bone Miner. Res..

[41] Lopez I., Aguilera-Tejero E., Mendoza F.J., Almaden Y., Perez J., Martin D., Rodriguez M. (2006). Calcimimetic R-568 decreases extraosseous calcifications in uremic rats treated with calcitriol. J. Am. Soc. Nephrol..

[42] Raggi P., Chertow G.M., Torres P.U., Csiky B., Naso A., Nossuli K., Moustafa M., Goodman W.G., Lopez N., Downey G. (2011). The ADVANCE study: A randomized study to evaluate the effects of cinacalcet plus low-dose vitamin D on vascular calcification in patients on hemodialysis. Nephrol. Dial. Transplant..

[43] Rojas M.G., Pereira-Simon S., Zigmond Z.M., Varona Santos J., Perla M., Santos Falcon N., Stoyell-Conti F.F., Salama A., Yang X., Long X. (2024). Single-Cell Analyses Offer Insights into the Different Remodeling Programs of Arteries and Veins. Cells.

[44] Jankovic A., Damjanovic T., Djuric Z., Marinkovic J., Schlieper G., Djuric P., Dragovic J.T., Bulatovic A., Mitrovic M., Popovic J. (2017). Calcification in arteriovenous fistula blood vessels may predict arteriovenous fistula failure: A 5-year follow-up study. Int. Urol. Nephrol..

[45] Zha Y., Qian Q. (2017). Protein Nutrition and Malnutrition in CKD and ESRD. Nutrients.

[46] Zyga S., Christopoulou G., Malliarou M. (2011). Malnutrition-inflammation-atherosclerosis syndrome in patients with end-stage renal disease. J. Ren. Care.

[47] Enia G., Sicuso C., Alati G., Zoccali C. (1993). Subjective global assessment of nutrition in dialysis patients. Nephrol. Dial. Transplant..

